# Quadruple Shape-Memory Organohydrogels with Adjustable Trigger Temperatures

**DOI:** 10.1021/acsomega.5c05082

**Published:** 2025-08-12

**Authors:** Esra Su, Cigdem Buse Oral, Oguz Okay

**Affiliations:** † Faculty of Aquatic Sciences, 37516 Istanbul University, Fatih, Istanbul 34134, Turkey; ‡ Department of Chemistry, 52971 Istanbul Technical University, Maslak, Istanbul 34469, Turkey

## Abstract

Organohydrogels (OHGs) are a class of soft materials with a biphasic structure consisting of hydrophilic and hydrophobic domains that interact with both water and organic solvents. This gives them unique properties and various applications, e.g., in biomedicine, antifreeze, soft robotics and environmental engineering, where adaptability and resilience are crucial. In the present work, we present OHG systems consisting of a continuous hydrogel phase based on silk fibroin (SF) in which single, binary or ternary combinations of semicrystalline poly­(n-tetradecyl acrylate) (PC14A), poly­(n-hexadecyl acrylate) (PC16A) and poly­(n-octadecyl acrylate) (PC18A) micro-organogels with side chain lengths between 14 and 18 are dispersed. The OHGs withstand 90–94% compression without failure and have a high Young’s modulus (up to 2.3 MPa) at room temperature. They exhibit thermosensitive viscoelastic and mechanical properties, and an effective shape-memory effect with finely adjustable trigger temperatures. We also show that the dimer or trimer combinations of hydrophobic poly­(*n*-alkyl acrylates) exhibit cocrystallization that hinder the multishape-memory behavior in OHGs. To overcome this difficulty, we developed a “gluing method” in which the three hydrophobic layers are bonded together without mixing to create microinclusions with three hydrophobic layers. In this way, we were able to produce OHGs with quadruple shape-memory behavior, which have trigger temperatures of 40, 30, and 15 °C.

## Graphical abstract

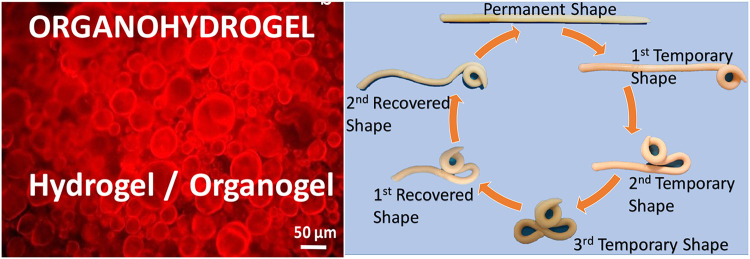

## 1 Introduction

Biological systems such as tissues contain both hydrophilic and hydrophobic components that give them extraordinary properties such as freezing tolerance, elasticity, and adaptive biomechanics.^1–4^ The concept of dynamic coexistence of opposing components in biological systems is very intriguing and has led to advances in materials science, particularly in the development of organohydrogels (OHGs) by combining two components with opposing physicochemical properties.^5–7^ The properties of OHG could potentially open up a range of applications, for example in biomedicine, antifreeze, soft robotics and environmental technology, where adaptability and resilience are critical.^8–13^

The synthesis of OHG typically employs strategies that take advantage of the unique properties of both hydrophobic and hydrophilic components. Two main techniques are used to produce these smart materials: In the oil-in-water emulsion method, a hydrophobic phase (e.g., oil) is dispersed in an aqueous solution to form an oil-in-water emulsion.^1,5^ Once the emulsion is stabilized, the aqueous phase undergoes gelation, creating a network in which the hydrophobic and hydrophilic components coexist. This method makes it possible to combine different properties in a single material by using both water-loving and water-repellent substances. The second technique is to incorporate hydrophobic components into hydrophilic networks. In this approach, hydrophobic components are incorporated into a preformed hydrophilic network.^12,14–16^ This method is particularly useful for the development of OHGs that must remain flexible and function in low temperature environments. Although these synthesis methods have been successful in producing OHGs with the desired properties, they mainly focus on the production of nonfreezable materials and neglect their response to external stimuli or changes in physical conditions. Another limitation is their poor mechanical properties, which limit their use in applications that require materials that can withstand significant mechanical stress or deformation. Improving the mechanical properties of OHG is critical to expanding their practical applications, particularly in areas such as bioengineering, soft robotics and medical devices.

Silk fibroin (SF) is one of the most important and widely used proteins in biomedical applications due to its excellent mechanical properties, biocompatibility and biodegradability.^17–19^ SF also functions as a natural emulsifier due to its amphiphilic multiblock copolymer character.^20^ Recently, we have shown that SF facilitates the formation of stable oil-in-water emulsions in the production of OHGs without the need for additional emulsifiers.^21,22^ This makes the process of producing OHGs more environmentally friendly and biocompatible. In addition, SF imparts stability to the emulsion by facilitating the formation of β-sheets, increasing their hydrophobicity, and serving as a physical cross-linker.^21^ SF-based OHG systems, which possess both hydrophilic and hydrophobic domains, consist of a continuous SF hydrogel or cryogel phase in which semicrystalline poly­(n-octadecyl acrylate) microinclusions (PC18A) are dispersed.^16,21^ (Figure 1a) Since PC18A was the only component in the oleophilic phase that could produce switchable mechanical and viscoelastic properties, the OHGs exhibited a biphasic transition below or above about 50 °C, the melting temperature of PC18A. However, changing the length of the alkyl side chains of poly­(alkyl acrylates) in the microinclusions would result in OHGs with shape-memory effects and tunable trigger temperatures, a property that expands their application as flexible electronic devices, smart medical devices, sensors and actuators, smart textiles, aerospace applications, and many others.^23^ Recently, we used a combination of PC18A and several long-chain hydrocarbons with different chain lengths as micro-organogels to prepare OHGs with multiple shape-memory.^22^ However, the hydrocarbons in the OHG dissolved when immersed in ethanol, and their shape-memory properties were lost over time.

**Figure 1:**
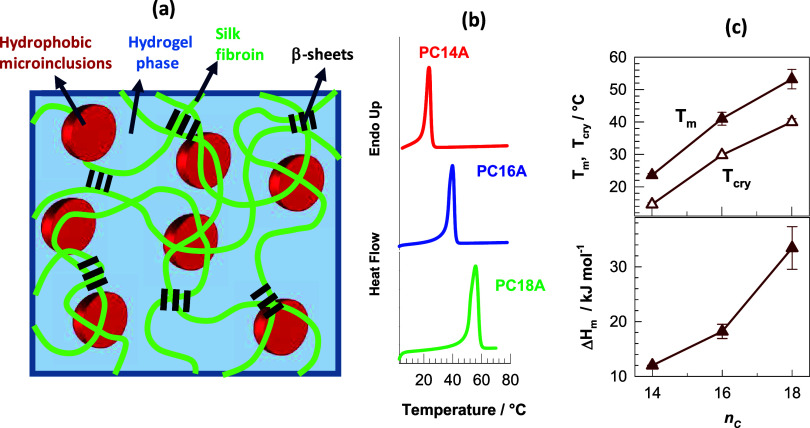
(a) Scheme of OHG consisting of a continuous SF-based hydrogel phase (light blue) in which semicrystalline hydrophobic microinclusions are dispersed (red). (b) Heating DSC scans of PC14A, PC16A, and PC18A. (c) The melting *T*_m_ and crystallization temperatures *T*_cry_, and melting enthalpies Δ*H*_m_ plotted against the number of carbon atoms on the side chains (*n*_C_) of the hydrophobes.

In the present work, we prepare OHGs containing poly­(n-tetradecyl acrylate) (PC14A), poly­(n-hexadecyl acrylate) (PC16A), and poly­(n-octadecyl acrylate) (PC18A) microinclusions with 14, 16, and 18 carbon atoms in the side chains (*n*_C_), respectively. The melting and crystallization temperatures of the hydrophobes increase from 23.6 ± 0.5 to 53 ± 3 °C and from 14.6 ± 0.5 to 40 ± 1 °C, respectively, when the number of carbon atoms on the side chains (*n*_C_) increases from 14 to 18 (Figure 1b,c). Their melting enthalpies Δ*H*_m_ also increases with *n*_C_, indicating an increasing degree of crystallinity (Figure 1c). OHGs containing single, binary or ternary combinations of these poly­(n-acrylates) are expected to exhibit a shape-memory effect with a trigger temperature that can be varied over a wide temperature range. As will be shown below, the OHGs exhibit thermosensitive viscoelastic and mechanical properties, and an effective shape-memory effect with finely tunable trigger temperatures. We also show that the dimer or trimer combinations of poly­(*n*-alkyl acrylates) with *n*_C_ = 14, 16, and 18 exhibit cocrystallization that hinder the multishape-memory behavior in OHGs. To overcome this difficulty, we developed a “gluing method” in which the three hydrophobic layers are bonded together without mixing to create microinclusions with three hydrophobic layers. In this way, we were able to produce OHGs with quadruple shape-memory behavior, which have trigger temperatures of 40, 30, and 15 °C.

## 2 Results and Discussion

### 2.1 OHGs with Dual Shape-Memory Effect

OHGs were prepared by first creating a stable oil-in-water emulsion without using an external emulsifier. For this purpose, the hydrophobic monomer, namely n-tetradecyl acrylate (C14A), n-hexadecyl acrylate (C16A) or n-octadecyl acrylate (C18A), is dispersed in an aqueous silk fibroin (SF) solution. To increase the stability of the emulsion over time, ethanol is added.^21^ Ethanol induces gelation in the continuous SF phase by triggering a conformational change in SF from a random coil structure to β-sheet structures, which imparts structural stability to the emulsion.^21^ In the second step, in situ polymerization of the hydrophobic monomer droplets within the emulsion is performed using UV light and a photoinitiator. This step leads to the formation of high-strength OHGs that exhibit switchable viscoelasticity and shape-memory properties. To further improve the mechanical strength and shape-memory properties of the OHGs, hydrophilic N,N-dimethylacrylamide units are incorporated into the hydrogel phase to optimize the functionality of the material.^21^

Figure 2a–c shows confocal laser scanning microscopy (CLSM) images of OHGs with PC18A (a), PC16A (b) and PC14A (c) in their microinclusions, where the dispersed hydrophobic phase was stained with Nile red. The individual hydrophobic domains with diameters between 5 and 150 μm are dispersed in the continuous hydrophilic phase. These domains act as microinclusions in the OHG and provide shape-memory properties and thermomechanical performance.

**Figure 2:**
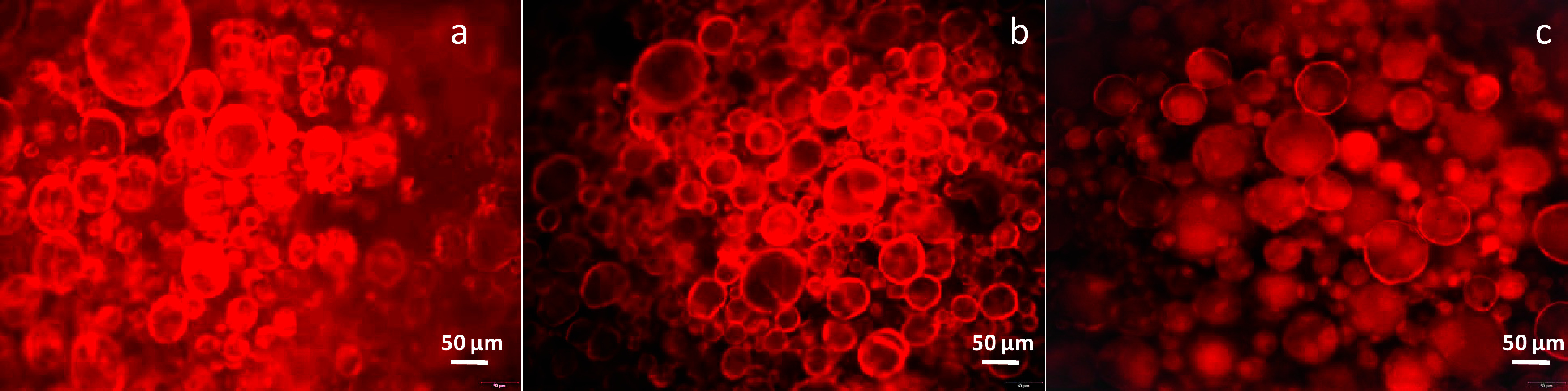
CLSM images of OHGs with PC18A (a), PC16A (b), and PC14A (c) in their microinclusions. The microinclusions were stained with Nile red. Scale bars: 50 μm.

The OHG exhibited a swelling ratio *m*_rel_ of about 1.2 in water, which increased slightly with decreasing number of carbon atoms (*n*_C_) on the side chain (Figure S1a). The gel fraction, i.e., the fraction of reactants incorporated into the OHG network, was 0.97 ± 0.02 and 1.07 ± 0.02 for water and ethanol, respectively, indicating almost complete conversion of the reactants into the OHG network (Figure S1b). The chemical structure of the OHGs in their dry state was investigated by FTIR spectroscopy (Figure 3). The characteristic peaks of PC14A, PC16A and PC18A in the microinclusion of OHGs can be seen in their spectra at 2920 and 2850 cm^–1^, which are due to the C–H stretching vibrations of methylene groups, and at 1730 and 1160 cm^–1^, which are due to the stretching vibrations of C=O and C–O–C, respectively. The peak at 1620 cm^–1^ is typical of the β-sheet structure of SF and indicates a conformational transition in SF from a random coil to a β-sheet structure and thus gelation in the continuous phase of OHGs.^22^ (see Supporting Information, Text S1, for details) Thus, all peaks observed in PC14A, PC16A and PC18A as well as in the SF hydrogel network appear in the spectra of OHGs, indicating that no chemical reaction occurs between the components.

**Figure 3:**
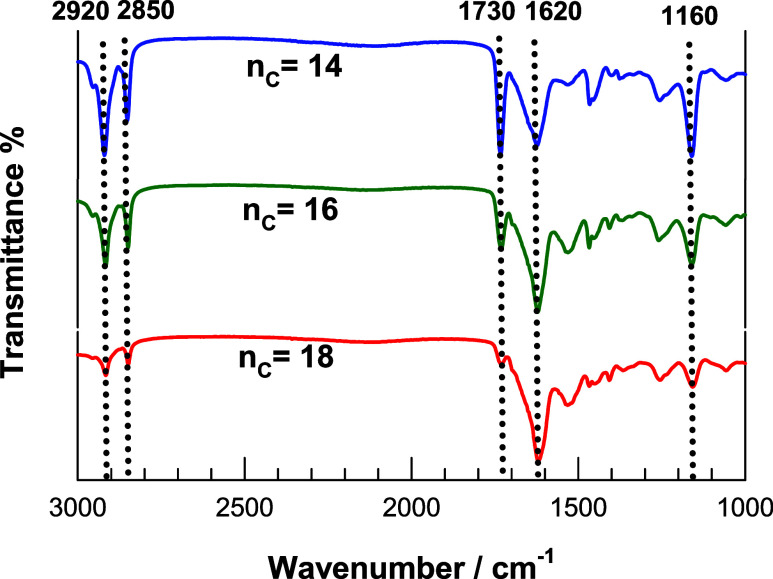
FTIR spectra of OHGs with PC14A, PC16A, and PC18A in their microinclusions.

The solid and dashed curves in Figure 4a show DSC scans upon heating and cooling of OHGs containing PC18A, PC16A, and PC14A in their microinclusions, respectively, while Figure 4b shows the *T*_m_ and *T*_cry_ temperatures of OHGs as a function of *n*_C_. The *T*_m_ values are 25 ± 1, 43 ± 2 and 53 ± 2 °C for *n*_C_ = 14, 16 and 18, respectively, close to the *T*_m_ values of the pure polymers (Figure 1c). Similar results were also obtained for the *T*_cry_ temperatures, indicating that the inclusion of the hydrophobic components in the continuous SF phase does not affect their thermal properties. Figure 4c shows XRD scans of OHG with *n*_C_ = 16 and 18, while the OHG with *n*_C_ = 14 could not be measured because its *T*_m_ (25 °C) was too close to room temperature. The OHGs exhibit a sharp peak at 21.5°, corresponding to a Bragg *d*-spacing of 0.41 nm, which is typical of the hexagonal packing of C16 and C18 side chains.^24^ Thus, both the DSC and XRD results show that the crystalline domains could be successfully generated within the microinclusions dispersed in the continuous SF hydrogel phase.

**Figure 4:**
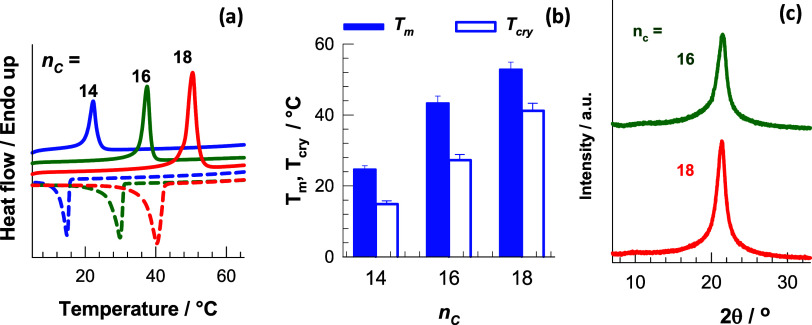
(a) Heating and cooling DSC scans of OHGs prepared using PC14A, P16A, and PC18A. (b) Melting *T*_m_ and crystallization temperatures *T*_cry_ of OHGs plotted against *n*_C_. (c) X-ray diffraction patterns of OHGs with *n*_C_ = 16 and 18.

The mechanical and viscoelastic properties of the OHGs changed significantly and reversibly with temperature due to the melting and crystallization of crystalline domains. Figure 5 shows the storage moduli *G′* (filled symbols) and the loss moduli *G*″ (open symbols) of OHGs subjected to a temperature cycle between 25 and 65 °C. The vertical dashed red and blue lines represent *T*_m_ and *T*_cry_ of the OHGs, respectively, as determined by DSC. At 25 °C, *G′* is 107, 450, and 610 kPa for OHGs with *n*_C_ = 14, 16 and 18, respectively, indicating an increasing modulus with increasing length of the alkyl side chains (Figure 6a). When the temperature is increased from 25 to 65 °C, the OHGs become weak and *G′* decreases to 40, 56, and 82 kPa for *n*_C_ = 14, 16 and 18, respectively, indicating a 2.6, 8.3 and 7.4-fold decrease in storage modulus due to melting of the crystalline domains. In addition, *G′* of the OHGs is fully recovered by recooling the sample to 25 °C. The loss modulus *G*″ exhibits a similar behavior, while the loss factor *tan δ* remains around 0.1 throughout the thermal cycle, indicating viscoelastic behavior of the OHGs (Figure S2). The results also show that the temperatures *T*_m_ and *T*_cry_ measured in the DSC agree with the results of the rheometer tests for OHGs with *n*_C_ = 16 and 18 and *tan δ* assumes a weak maximum. However, melting and crystallization of OHG with *n*_C_ = 14 occur later, i.e., at a higher temperature in the rheometer compared to that in the DSC scans, e.g., *T*_m_ = ∼32 versus 25 °C and *T*_cry_ = ∼31 versus 15 °C for rheometer and DSC. This is due to the larger sample volume in the rheometer compared to the DSC, so a longer period of time is required to reach thermal equilibrium.

**Figure 5:**
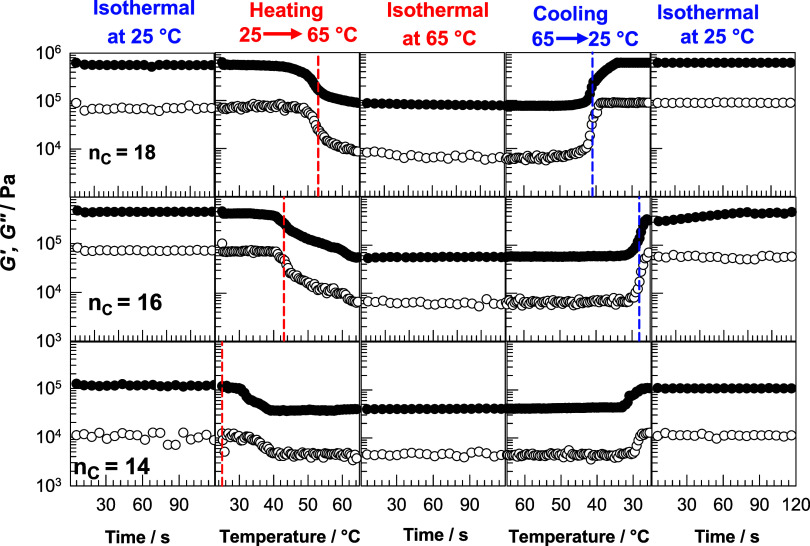
*G′* (filled symbols) and *G*″ (open symbols) of OHGs with *n*_C_ = 14, 16, and 18 subjected to a thermal cycle between 25 and 65 °C. The vertical dashed red and blue lines represent *T*_m_ and *T*_cry_ of the OHGs, respectively, as determined by DSC. γ_*o*_ = 0.1%. ω = 6.28 rad·s^–1^.

**Figure 6:**
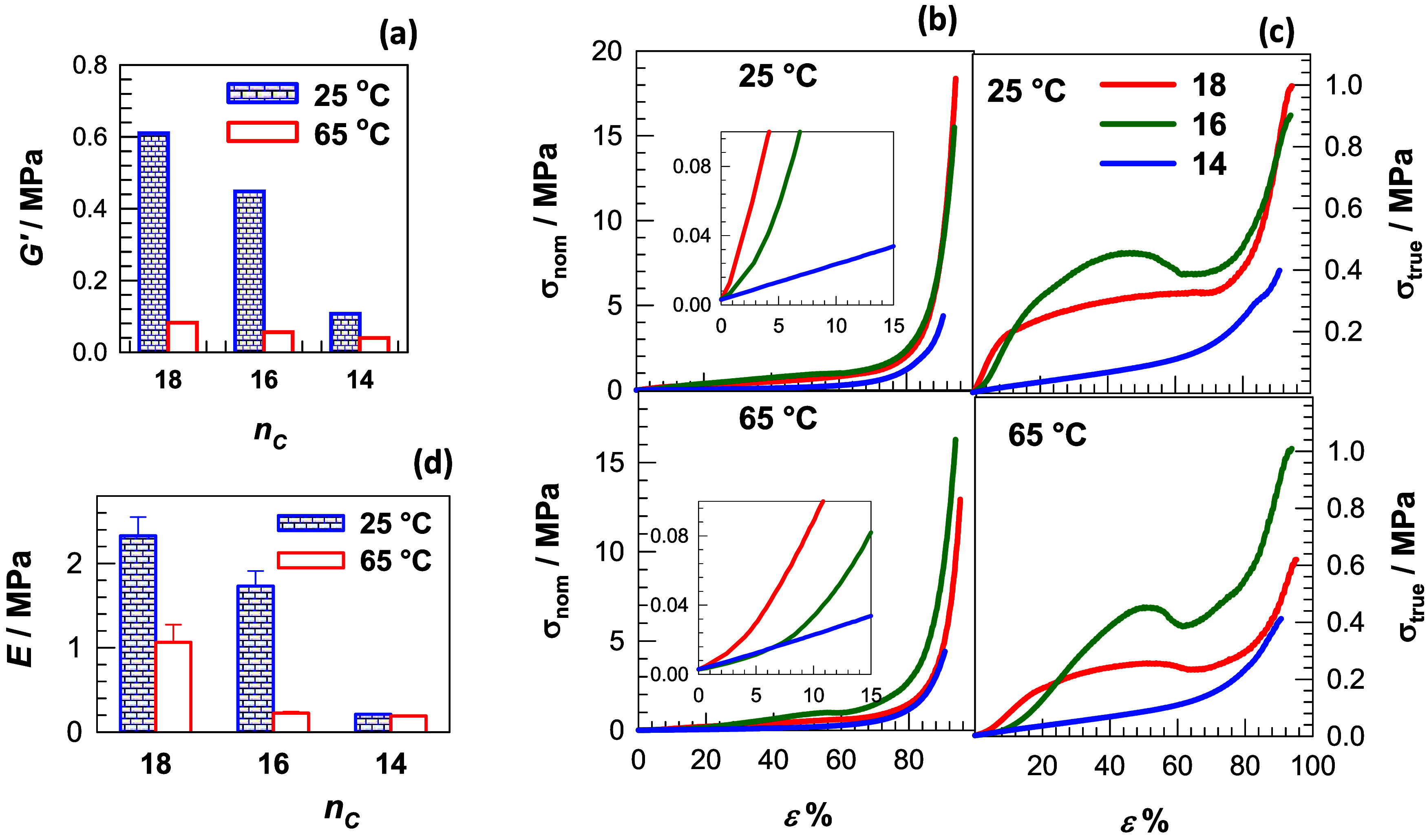
(a) Storage modulus *G′* of OHGs at 25 and 65 °C. (b, c) Nominal σ_nom_ and true stresses σ_true_ plotted against strain ε for OHGs containing PC18A, PC16A, and PC14A in their microinclusions, indicated as 18, 16, and 14, respectively. (d) Young’s modulus *E* of OHGs at 25 and 65 °C plotted against *n*_C_.

The mechanical properties of OHG were investigated by uniaxial compression tests on water-swollen OHG samples at 25 and 65 °C, i.e., below and above the *T*_m_ of OHG, respectively. The data are presented as nominal stresses σ_nom_ and true stresses σ_true_, i.e., the forces acting on the undeformed and deformed cross-sectional area of the specimens, respectively, plotted against the strain ε (see Supporting Information, Text S2, for details). We should mention that the true stress σ_true_ is more realistic as it indicates the force acting on the actual surface of the OHG specimens.^25^ Figure 6b, c shows σ_nom_ – ε (b) and σ_true_ – ε curves (c) of OHGs at 25 (upper panel) and 65 °C (bottom panel). All OHGs withstand 90–94% compressive load without failing. Young’s modulus *E* and the fracture stress σ_*f*_ of the OHGs decrease with decreasing *n*_C_, e.g., *E* is 2.3 ± 0.2, 1.7 ± 0.2 and 0.2 ± 0.01 MPa for *n*_C_ = 18, 16 and 14, respectively, which is due to the simultaneous decrease in crystallinity of the OHGs (Figure 6d). The modulus *E* also decreases when the temperature is increased from 25 to 65 °C, which is due to the melting of the alkyl crystals. In addition, the curves σ_true_ – ε show the occurrence of yield points for OHG with *n*_C_ = 18 and 16, while no yield behavior occurs for the low crystalline OHG with *n*_C_ = 14. The yield behavior can be explained by the disintegration of the crystalline blocks at the yield point, which leads to an energy dissipation mechanism in which the amorphous regions hold the macroscopic sample together.^26^

The shape-memory behavior of OHG is investigated by means of bending tests on cylindrical, water-swollen OHG samples. Figure 7a shows the shape fixity ratio *R*_f_ of OHGs with fixation temperatures of 4 and 25 °C. The OHG with *n*_C_ = 14 exhibits a low shape fixity ratio of 68% at 25 °C due to the low degree of crystallization, while *R*_f_ increases to 92% when the fixation temperature is lowered from 25 to 5 °C. The other OHGs with larger hydrophobes show an almost complete shape fixity ratio at both 25 and 5 °C. In Figure 7b, the shape recovery ratio *R*_*r*_ of the OHGs is plotted against temperature. All OHGs show a high shape-recovery of over 80%. The recovery of the permanent shape from the temporary shapes starts at 26, 37, and 51 °C for *n*_C_ = 14, 16 and 18, respectively, which are close to their melting temperatures (Figure 4b). Moreover, the recovery of the permanent shape of OHGs at 65 °C occurs within 6 min, and the initial recovery rate decreases with increasing *n*_C_, i.e., with increasing crystallinity of OHGs due to the longer fixation of the temporary shape (Figure S3).

**Figure 7:**
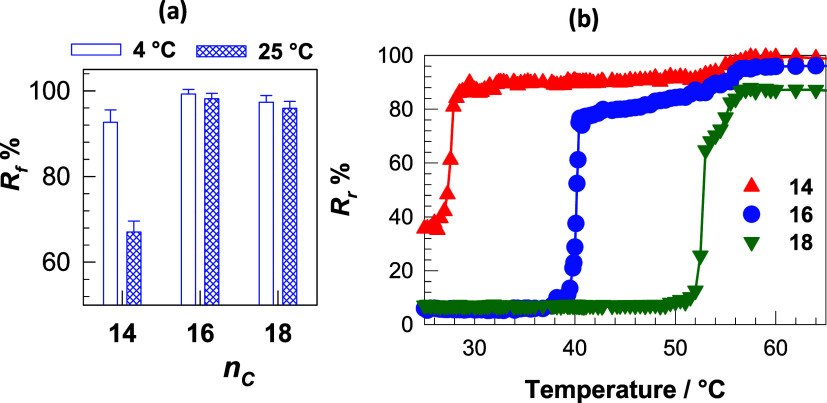
(a) Shape-fixity ratio *R*_f_ of OHGs at 4 and 25 °C plotted against *n*_C_. (b) Temperature dependence of the shape-recovery ratio *R*_r_ of OHGs with various *n*_C_ as indicated.

In addition to the dual shape-memory behavior of OHGs, they also show freezing tolerance and thus elasticity at subzero temperatures, as reported before for several OHGs.^4–6,12–14,27,28^ Freeze tolerance of OHG was achieved by coating it with a thin layer of glycerol, which is known to lower the freezing point of water due to the strong H-bonds between glycerol and water, which destroy ice crystals at low temperatures.^29,30^ For example, Figure 8a shows DSC scans of OHGs without (left) and with glycerol (right), which show that freezing down to −50 °C cannot be observed when coated with glycerol. Figure 8b shows the elasticity of two OHG samples without and with glycerol coating. After coating, the sample can be easily deformed into a horseshoe shape at −20 °C without failure, while the sample without coating breaks at the same temperature.

**Figure 8:**
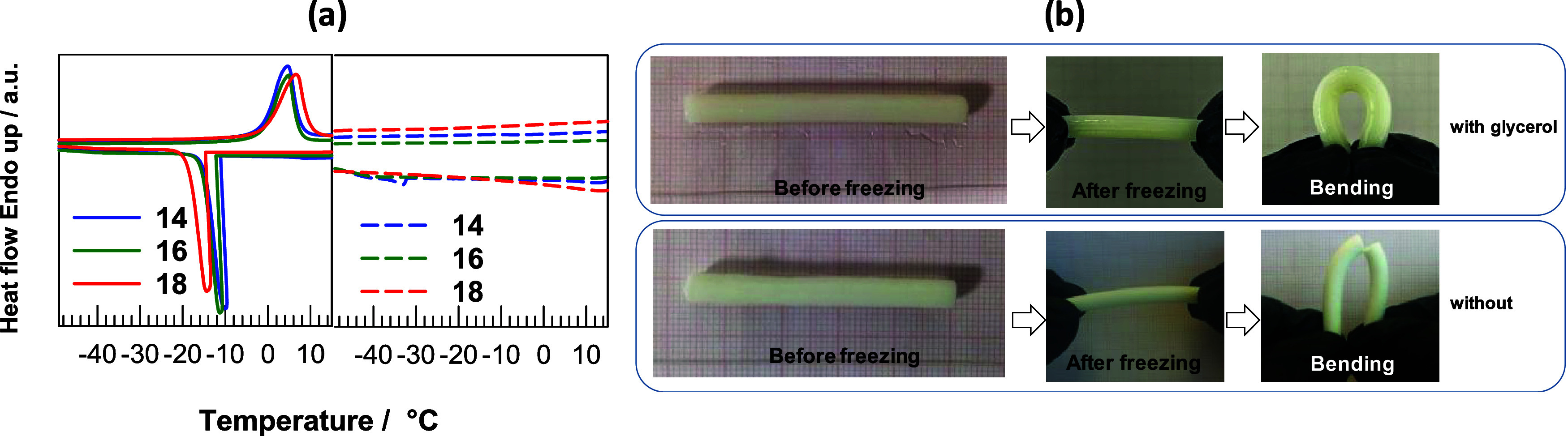
(a) Heating and cooling DSC scans of OHGs without (left) and with glycerol (right). (b) Two OHG specimens without and with coated glycerol at −20 °C. The coated specimen can easily be deformed to a horseshoe shape at −20 °C without any failure (upper panel) while that without coating ruptures at the same temperature.

### 2.2 OHGs with Quadruple Shape-Memory Effect

To create a quadruple shape-memory effect in OHGs, the hydrophobic copolymers in the microinclusions should have different phase transition temperatures when they are mixed together. To verify this, the thermal behavior of the hydrophobic copolymers was investigated by polymerizing dimer and trimer combinations of C14A, C16A, and C18A monomers in the presence of Irgacure photoinitiator to form P­(C18A-*co*-C16A), P­(C18A-*co*-C14A), and P­(C18A-*co*-C16A-*co*-C14A) polymers. The DSC scans of the copolymers at different compositions are shown in Figure 9a. The copolymers exhibit single *T*_m_ or *T*_cry_ temperatures, indicating cocrystallization. This behavior differs from the behavior of PC18A/*n*-alkane mixtures, which form noneutectic mixtures over a wide range of compositions,^22^ indicating that the polymer mixtures facilitate cocrystallization, which is due to the lower mobility of two alkyl side chains on different chains compared to one alkyl chain with alkanes. In addition, the position of the peak observed at 53 °C for pure PC18A shifts toward the peak of pure PC16A (41 °C) or PC14A (24 °C) with increasing PC16A or PC14A content.

**Figure 9:**
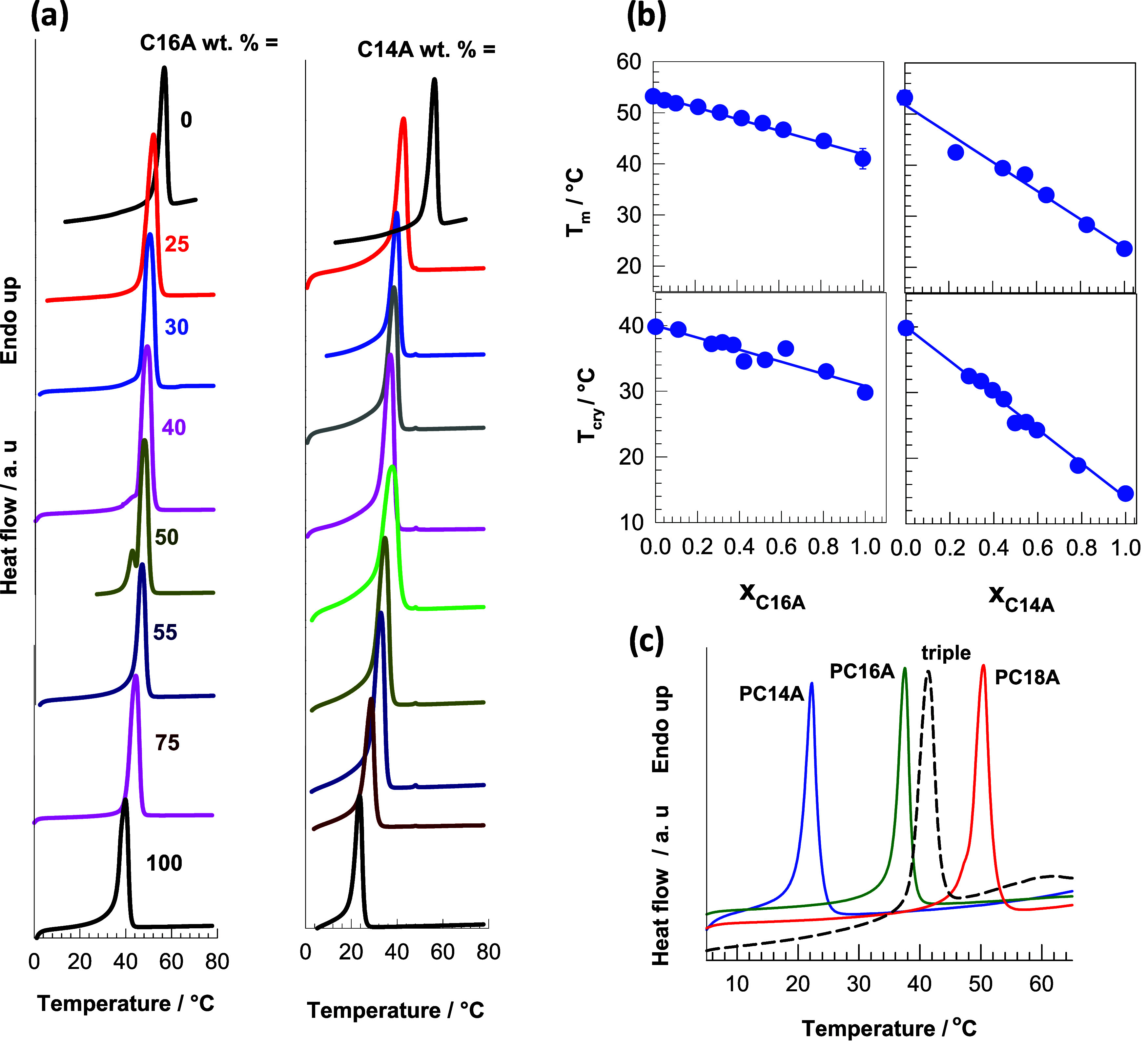
(a) Heating DSC scans of P­(C18A-*co*-PC16A) (left panel) and P­(C18A-*co*-PC14A) (right panel) at various compositions as indicated. (b) Melting *T*_m_ and crystallization temperatures *T*_cry_ of copolymers plotted against the mole fraction of PC16A or PC14A. The lines are calculated using eq 1a, b. (c) Heating DSC scan (dashed curve) of OHG containing P­(C18A-*co*-C16A-*co*-C18A) in equal amounts while the solid curves are DSC scans of OHGs with pure PC14A, PC16A, and PC18A in their microinclusions.

Figure 9b showing the compositional dependence of *T*_m_ and *T*_cry_ of the copolymers indicates a linear decrease in transition temperatures with increasing mole fraction of C16A or C14A. Assuming that the mole fractions of the polymer components determine the transition temperatures of the copolymers, *T*_m_ and *T*_cry_ can be calculated using the following equations,

1a $$T_{m}=\sum T_{m,i}x_{i}$$

1b $$T_{\mathrm{cry}}=\sum T_{\mathrm{cry},i}x_{i}$$

where T_m,*i*_ and x_*i*_ are the melting temperature and the mole fraction of component *i* in the copolymers, respectively. The solid curves in Figure 9b calculated using eq 1a, b fit well with the experimental *T*_m_ and *T*_cry_ data, indicating that the C16A and C14A units cocrystallize with the C18A units and form single alkyl crystals regardless of the composition of the copolymer. An important point of this finding is that the trigger temperature of the hydrophobes and thus the OHG can be precisely adjusted between 53 and 23 °C by varying the copolymer composition (Figure 9b). The melting enthalpies Δ*H*_m_, in kJ per mole of the repeating units, of the hydrophobic copolymers also decrease with increasing mole fraction of C16A or C14A units, reflecting the decreasing degree of crystallization (Figure S4). The dashed curve in Figure 9c shows a DSC scan of OHG containing triple combinations of PC14A, PC16A and PC18A in equal amounts, while the solid curves represent DSC scans of OHG with pure PC14A, PC16A and PC18A in microinclusions. The OHG with triple hydrophobes again exhibits a single *T*_m_ and *T*_cry_ at 41 ± 2 and 30 ± 2 °C, respectively, indicating cocrystallization. These values are close to the theoretical values calculated using eq 1a, b, e.g., *T*_m_ = 40 ± 2 °C and *T*_cry_ = 28 ± 2 °C, indicating the formation of single crystals.

The results thus show that the mixtures of poly­(*n*-alkyl acrylates) with *n*_C_ = 14, 16, and 18 exhibit cocrystallization that prevent multishape- memory behavior. To overcome this obstacle, we developed a “gluing method” in which the three hydrophobes are bonded together without mixing. To this end, we first prepared three emulsions containing the same aqueous SF solution but different hydrophobic monomers, namely C14A, C16A and C18A, and a photoinitiator dispersed in the continuous SF solution. The first emulsion containing C18A was transferred to a plastic pipet (reactor, 200 mm length, 10 mm diameter) and kept in the UV reactor for 1 min until the emulsion was no longer liquid to prevent interdiffusion of the layers. The second emulsion containing C16A was then slowly added to the surface of the pipet and again left in the UV reactor for 1 min. The same procedure is repeated with the addition of the third emulsion containing C14A to form an emulsion consisting of three layers. The hybrid OHGs with layered hydrophobes showed their individual thermal properties with a smooth and robust interface between them. Figure 10a shows an OHG sample with three hydrophobic regions with *n*_C_ = 14, 16 and 18, while the red region indicates the interfacial regions between C14/C16 and C16/C18. The DSC heating scans of the hydrophobic and interfacial regions of the OHG are shown in Figure 10b,c, respectively. It can be seen that *T*_m_ and *T*_cry_ in the layers are the same as in the pure hydrophobic components, while in the interface region there is a smooth transition from one layer to the other.

**Figure 10:**
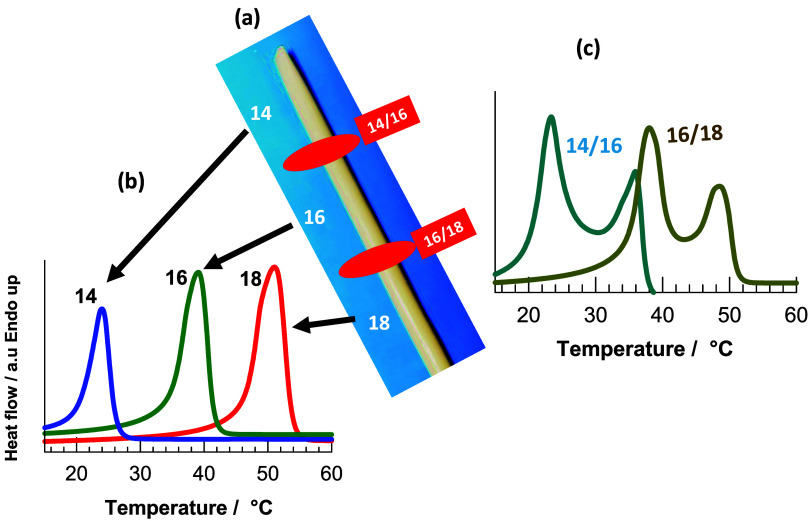
(a) An OHG specimen with three hydrophobic regions with *n*_C_ = 14, 16, and 18. The red area indicates the interface regions between *n*_C_ = 14/16 and 16/18. (b, c): Heating DSC scans of hydrophobic regions with *n*_C_ = 14, 16, and 18 (b), and intersection regions 14/16 and 16/18 of the same specimen (c).

The presence of three-phase transition temperatures led to a quadruple shape-memory effect in OHGs, as demonstrated by a rectangular-shaped OHG sample immersed in water (Figure 11). The sample in the permanent shape was first heated to 65 °C, which is above the *T*_m_ values of the hydrophobic components. At this temperature, it is in the amorphous state so that the sample can be easily deformed. After deformation of the sample and subsequent cooling to 40 °C under tension, i.e., between the *T*_m_ values of PC18A and PC16A, the first temporary shape is fixed (Figure 11). By deforming the sample again at 40 °C and then cooling it to 30 °C, i.e., between the *T*_m_ values of PC16A and PC14A, the second temporary shape is fixed. By repeating these steps with deformation at 30 °C and subsequent cooling to 15 °C, i.e., below the *T*_m_ values of the components, the third temporary shape was fixed. To restore the permanent shape, the sample is gradually heated to 65 °C, which causes a gradual melting of the crystalline regions and ensures that the chains return to their most entropically favorable initial conformation. Cyclic shape memory tests show reversible recovery of the permanent shape of OHG specimens (see Supporting Information, Movie). This was to be expected, as the storage and loss moduli of OHG change reversibly during thermal cycling (Figure 5). The result thus shows that the “gluing method” can be used to create triple, quadruple, quintuple, and sextuple shape memory OHGs by increasing the number of interconnected layers in the OHGs to 3, 4, 5, and 6.

**Figure 11:**
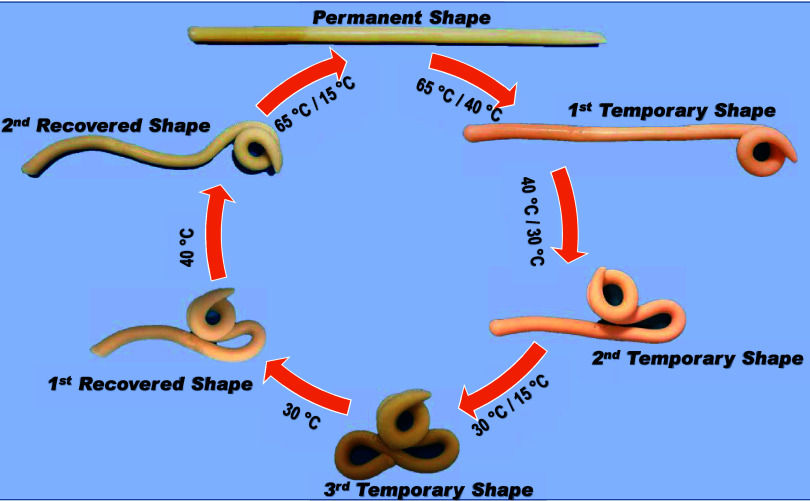
Quadruple shape-memory behavior of an OHG specimen with layered hydrophobes.

## 3 Conclusions

The ability of OHGs to mimic the synergistic biphasic properties of biological systems offers an exciting opportunity for the development of advanced materials with special properties. Here, we have developed innovative OHGs that feature a stable SF hydrogel as a continuous phase in which hydrophobic, semicrystalline PC14A, PC14A or PC18A polymers with carbon chain lengths of 14, 16 and 18, respectively, are dispersed. By changing the chain length of the polymers, a range of OHGs with adjustable transition temperatures could be obtained to suit different applications. The hydrophobic polymers in the microinclusions of the OHG melt and crystallize at their respective phase transition temperature, leading to a gradual change in viscoelastic and mechanical properties. The OHGs also exhibit an effective shape-memory effect with adjustable trigger temperatures. We also show that the dimer or trimer combinations of hydrophobic poly­(*n*-alkyl acrylates) with *n*_C_ = 14, 16, and 18 exhibit cocrystallization that hinder the multishape-memory behavior in OHGs. To overcome this difficulty, we developed a “gluing method” in which the three hydrophobic layers are bonded together without mixing to create microinclusions with three hydrophobic layers. In this way, we were able to produce OHGs with quadruple shape-memory behavior, which have trigger temperatures of 40, 30, and 15 °C.

## 4 Materials and Methods

### 4.1 Materials

Cocoons of *Bombyx mori* silkworms (Kozabirlik, Agriculture Sales Cooperative for Silk Cocoon, Bursa, Turkey), sodium carbonate (Na_2_CO_3_, Merck, 99.9%), lithium bromide (LiBr, Merck, 99%), polyethylene glycol (PEG-10000, Sigma-Aldrich, 10,000 g·mol^–1^), ethanol (Merck, ≥ 99.9%), N,N′-methylenebis­(acrylamide) (BAAm, Sigma-Aldrich, 99%), n-tetradecyl acrylate, (C14A, Sigma-Aldrich, 95%), n-hexadecyl acrylate (C16A, Sigma-Aldrich, 98%), n-octadecyl acrylate (C18A, Sigma-Aldrich, 97%), 2-hydroxy-4′-(2-hydroxyethoxy)-2-methylpropiophenone (Irgacure 2959, Sigma-Aldrich, St. Louis, MO), and N,N-dimethylacrylamide (DMAA, Sigma-Aldrich, 99%) were used as received. Microinclusions of OHG were stained using Nile red (Sigma-Aldrich) and visualized.

### 4.2 Isolation and Preparation of SF^31^

*Bombyx mori* silkworm cocoons weighing around 10 g were broken up into tiny bits. The cocoon parts were cleaned with distilled water and then cooked for 1 h in a 0.02 M Na_2_CO_3_ solution (1 L) to remove sericin. To get rid of any remaining sericin and Na_2_CO_3_, the degummed SF was rinsed five times with 1 L of distilled water at 70 °C for 20 min each. The washed SF was dried at room temperature for 2 days. Seven g of the dried SF was dissolved in 35 mL of a 9.3 M LiBr solution at 60 °C for 2 h. LiBr effectively dissolves the SF by breaking down its crystalline structure, making it soluble. The SF solution was then dialyzed against water using a 10,000 MWCO dialysis tubing (Snake Skin, Pierce) for 3 days. The water was changed three times a day to ensure complete removal of LiBr. After dialysis, the solution was centrifuged to remove any undissolved particles, ensuring a homogeneous SF solution. The final SF concentration was found to be approximately 5 wt %. This was determined by weighing the remaining solid after drying a known volume of the SF solution. To prepare SF solutions with higher concentrations, the 5 wt % SF solution was further concentrated by dialysis against aqueous solutions of 15 w/v % PEG-10000 using 3,500 MWCO dialysis tubing (Snake Skin, Pearce). PEG acts as an osmotic agent, drawing water out of the SF solution and thereby increasing the concentration of SF. All SF solutions were stored at 4 °C to prevent microbial growth and degradation. The solutions were used within 2 weeks to ensure freshness and consistency in experimental conditions. A stock solution with a concentration of 9.51 w/v% SF was prepared and used for subsequent experiments.

### 4.3 Preparation of Dimer and Trimer Combinations of PC14A/PC16A/PC18A blends

To determine the conditions for achieving different phase transition temperatures in the microinclusions of OHG, dimer combinations of C14A/C18A and C16A/C18A monomers at various compositions as well as trimer combination of C14A/C16A/C18A at an equal mole ratio were mixed. The polymerization was then conducted in the presence of Irgacure 2959 (0.2 mol % with respect to the monomers) in a UV reactor (Kerman, Turkey) at 23 ± 2 °C at a wavelength of 360 nm for 24 h.

### 4.4 Synthesis of OHGs

The aqueous phase of OHG precursors was prepared by dissolving DMAA, BAAm, and Irgacure 2959 at 40 °C in the aqueous stock SF solution containing ethanol. Throughout this study, the subsequent parameters were maintained as they were the most effective means of achieving a stable emulsion of hydrophobic droplets measuring 12 ± 4 μm in diameter:^21^ SF = 6.5 w/v%; oil-to-water volume ratio = 1:1. DMAA = 7.5 w/w. Ethanol = 17 vol %. BAAm and Irgacure 2959 = 1 and 0.2 mol %, respectively, with respect to DMAA. The oil phase consisted of hydrophobic monomers at various combinations, and Irgacure 2959 (0.2 mol % of C18A). The oil phase was gradually added into the aqueous phase drop by drop while being continuously stirred for 5 min at 40 °C and 1400 rpm. The milky white emulsion was then transferred into plastic syringes of 1 and 50 mL in volumes and they were placed into the UV reactor at 23 ± 2 °C for 24 h. Typically, to prepare OHGs containing C16A in the microinclusions, 3.42 mL of aqueous SF stock solution and 0.36 mL of distilled water were mixed, and 0.84 mL of ethanol was dropwise added to this solution. After stirring the aqueous phase for 1h at 40 °C and 100 rpm, DMAA (0.39 mL), BAAm (5.83 mg) and Irgacure 2959 (1.69 mg) were added. After 1 h of stirring time, the oil phase consisting of C16A (5 mL) containing Irgacure 2959 (2.8 mg) was dropwise added to the aqueous phase while being continuously stirred for 5 min at 40 °C and 1400 rpm. The UV polymerization was then conducted at 23 ± 2 °C for 24 h. After polymerization, OHG specimens were placed in an excess of water for 3–4 days by replacing water every day to remove the soluble species, and to attain the swelling equilibrium. A part of the OHG specimens were dried in a vacuum oven (Nucleus, NVE) at 37 °C for 3–4 days to determine the gel fraction and swelling ratio. All other measurements including rheological, mechanical, DSC, and shape-memory tests were conducted on OHGs in their equilibrium swollen states in water.

### 4.5 Characterization

The secondary structure of SF was ascertained by means of attenuated total reflectance Fourier transform infrared spectroscopy (ATR-FTIR) observations on an Agilent Technologies Cary 630 ATR-FTIR spectrophotometer. A PerkinElmer DSC 4000 device was used to perform differential scanning calorimetry (DSC) measurements in a nitrogen atmosphere. Each specimen was weighed and placed in aluminum pans containing about 10 mg. Following that, the samples were scanned across a temperature range of 0 to 65 °C. The area under the endothermic peaks in the DSC curves was used to compute the melting enthalpies, Δ*H*_m_. X-ray diffraction (XRD) measurements were performed on dried specimens on a PANalytical X-Pert PRO multipurpose diffractometer using Ni-filtered Cu Kα radiation with a wavelength (λ) of 0.15418 nm at 45 kV and 40 mA in a 2θ range of 5 to 40°. Confocal laser scanning microscopy (CLSM, Leica TCs-SPE, Japan) was employed to visualize the distinct organogel component of OHGs by using Nile red stain. The equilibrium swelling ratio *m*_rel_ of OHGs in water was calculated as *m*_rel_ = *m*/*m*_o_ where *m* and *m*_o_ are the masses of equilibrium swollen and as-prepared OHGs, respectively. Moreover, the gel fraction *W*_g_ in water and ethanol was calculated using the equation

2 $$W_{g}=\frac{m_{\mathrm{dry}}}{m_{o}C_{o}}$$

where *m*_o_ is the mass of OHG after preparation, and *C*_o_ is concentration of SF, DMAA, BAAm, and hydrophobic PC18A, PC16A, or PC14A in the emulsion system.

Rheological tests were conducted by a Bohlin Gemini 150 Rheometer equipped with a Peltier device to precisely control the temperature. A parallel plate geometry with a diameter of 20 mm, and a water trap to prevent the evaporation of water from samples were used. Water-swollen OHG specimens prepared in 20 mL plastic syringes with an internal diameter of 20 mm were used for the measurements. Frequency (ω) sweep tests were carried out at a strain γ_o_ of 0.1%. The dependences of the storage (*G′*) and loss moduli (*G*″), and loss factor *tan δ* (= *G*″/*G′*) of OHGs on temperature were investigated by heating the OHG specimens from 25 to 65 °C at a rate of 4 °C·min^–1^ at γ_o_ = 0.1%, and ω = 6.28 rad·s^–1^.

Mechanical properties of water-swollen OHGs were characterized at 23 ± 2 °C using a Zwick-Roell universal test machine equipped with a load cell of 500 N. The tests were performed with a 0.05 N preload at a speed of 5 mm·min^–1^ in order to guarantee full contact between the sample and the surface. Young’s modulus *E* was calculated based on stress–strain curves between 2 or 4% compression.

The shape-memory behavior of OHG is investigated by means of bending tests on cylindrical, water-swollen OHG samples. For this purpose, an OHG sample is first heated above the *T*_m_ value of the crystalline areas, whereby it softens so that it can be easily bent into a horseshoe shape with a bending angle of 180° under the application of force. The sample was then cooled under force to a temperature below *T*_m_, and the force was then removed. The shape fixity ratio *R*_f_, which represents the ability of the specimen to fix the temporary shape, was calculated as^32,33^

3 $$R_{f}=\frac{\theta_{\mathrm{fix}}}{\theta_{\max}}$$

where θ_fix_ is the angle after removal of the force and after cooling and θ_max_ is the deformed angle under force, which is 180°. Another parameter that determines the efficiency of shape-memory is the shape recovery ratio *R*_r_, which is a measure of how well the sample returns to its original (permanent) shape after deformation. It can be quantified as a percentage of the original shape that is restored, i.e,

4 $$R_{r}=1-\frac{\theta_{r}}{\theta_{\max}}$$

where θ_r_ is the restored angle. Cyclic shape-memory properties of OHGs are studied by performing two shape memory cycles for the OHG with three hydrophobic regions with *n*_C_ = 14, 16, and 18. For this purpose, an OHG sample is first heated to 65 °C in a water bath to melt the crystalline domains. The softened sample was then formed into a spiral shape and cooled to 4 °C to fix the temporary spiral shape. The sample was then immersed in a water bath at 65 °C, during which a video recording of the sample was made. Two consecutive video recordings show the reversible recovery of the permanent shape of the OHG sample. To examine the antifreeze property of OHGs, the samples were cut into cylindrical shapes (about 15 mg) and immersed in glycerol to form a layer on the surface. They were then cooled and heated on DSC between 20 and −60 °C at a rate of 5 °C·min^–1^, and this process was repeated twice. The measurements were also conducted without coating with glycerol.

## Supplementary Material





## Notes

Microstructure of OHG components, relationship between nominal and true stresses, swelling ratio, gel fraction, rheological, time-dependent shape-recovery, and melting enthalpy data (PDF)
Movie showing cyclic shape-memory behavior (ZIP)

## §

Department of Chemistry, University of Manchester, Oxford Road, Manchester M13 9P2, UK
