# Supplementary material for: Quadruple Shape-Memory Organohydrogels with Adjustable Trigger Temperatures
Source: ACS Omega. 2025 Aug 12;10(33):37950–60. doi: 10.1021/acsomega.5c05082 (PMC12392180; doi:10.1021/acsomega.5c05082)
Supplement: Supplementary file 1 [file ao5c05082_si_001.pdf]

Supporting Information

Quadruple Shape-Memory Organohydrogels with Adjustable Trigger Temperatures

Esra Su[1,2*], *Cigdem Buse Oral*[2 +], *Oguz Okay*[2 *]

[1] Faculty of Aquatic Sciences, Istanbul University, Fatih, Istanbul 34134, Turkey

[2] Department of Chemistry, Istanbul Technical University, 34469 Maslak, Istanbul, Turkey

[+] Present address. Department of Chemistry, University of Manchester, Oxford Road, Manchester, M13 9P2 UK

Table of Contents

Text S1. Microstructure of OHG components before and after polymerization. S2

Text S2. The relationship between σ_{nom} and σ_{true}. S2

Figure S1. (a) Relative weight swelling ratio m_{rel} of OHGs plotted against n_C. (b) Gel fraction Wg of OHGs in water and in ethanol plotted against n_C. S3

Figure S2. Storage G' and loss moduli G'', and loss factor $\tan\delta$ of OHGs containing PC14A, PC16A, and PC18A micro-inclusions subjected to a thermal cycle between 25 and 65 °C. γ_o = 0.1%. ω = 6.28 rad·s^{-1}. S3

Figure S3. Time-dependent shape-recovery ratio R_r of OHG's with various n_C as indicated. Temperature = 65 °C S4

Figure S4. Composition dependence of the melting enthalpies ΔH_m of P(C18A-co-C16A) (a) and P(C18A-co-C14A) copolymers (b). The solid lines are predicted values using the equation $\Delta H_m = \sum \Delta H_{m,i}\, x_i$, where $\Delta H_{m,i}$ and x_i are melting enthalpy and the mole fraction of component *i* in the copolymer, respectively. S4

References S4

Text S1. Microstructure of OHG components before and after polymerization

We have previously examined the Amide I region of the FTIR spectra of the aqueous SF solution before and after the addition of ethanol and of the emulsion system before and after polymerization.[S1] Before polymerization, all samples show a sharp peak at 1640 cm^{-1} corresponding to the random coil conformation. After polymerization, the peak at 1640 cm^{-1} shifts to 1620 cm^{-1}, indicating a conformational transition in SF from the random coil to the β-sheet structure.[S1] The XRD pattern of SF before gelation shows a broad peak at 21°, corresponding to a random coil conformation.[S1] After gelation, the SF hydrogel component of OHG exhibits an intense peak at 20.6° and two weak peaks at 8.6 and 24.5°, corresponding to β-sheet crystal spacings of 4.3, 10, and 3.7 Å, respectively. Moreover, the XRD peaks of PDMAA at 10.5° and 22.8° overlap with the characteristic peak of SF at about 20°, which is due to the β sheet structure.[S2] Therefore, the peaks of PDMAA and SF cannot be distinguished based on XRD curves. In addition, the dispersed PC18A component of OHG shows a crystalline peak at 21.3°, corresponding to a Bragg d-spacing of 4.2 Å, which is typical of the paraffin-like hexagonal lattices formed by the packing of octadecyl (C18) side chains.[S1] The same peak also appears in the spectrum of OHG, indicating that the C18A droplets in the emulsion system have polymerized and formed crystalline domains. Besides this peak, second-order diffraction peaks appear at 25.3, 23.5, and 19.6°, which we attribute to the crystalline β-sheet structure.

Text S2. The relationship between σ_{nom} and σ_{true}

The compressive stress was represented by its nominal σ_{nom} and true values σ_{true}, which are the forces per cross-sectional area of the original and the deformed gel specimens, respectively. Thus, σ_{nom} and σ_{true} are given by

$$\rho_{nom} = \frac{l}{A_o} \quad \text{(S1)}$$

$$\rho_{true} = \frac{l}{A} \quad \text{(S2)}$$

where l is the sample length under force, A_o and A are the cross-sectional area of the original and the deformed gel specimens, respectively. Assuming isotropic deformation during compression, i.e., assuming that the gel volume remains constant during deformation, one obtains,

$$A_o\, l_o = A\, l \quad \text{(S3)}$$

$$\frac{A_o}{A} = \frac{l}{l_o} = \lambda \quad \text{(S4)}$$

where l_o is the initial length of the sample, and λ is the deformation ratio. From equations S1-S4, the relationship between σ_{nom} and σ_{true} is as follows:[S3]

$$\rho_{true} = \rho_{nom}\, \lambda \quad \text{(S5)}$$

Moreover, the change in the sample length ε (= $\Delta l/l_o$) relates to λ by $\varepsilon = \lambda - 1$.

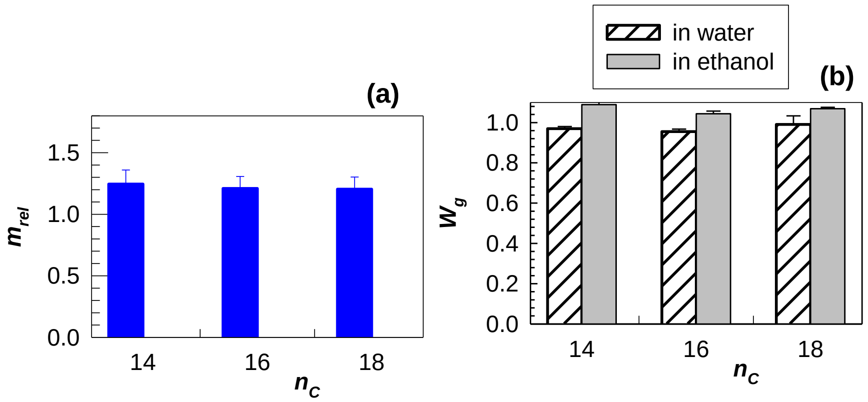

Figure S1. (a) Relative weight swelling ratio m_{rel} of OHGs plotted against n_C. (b) Gel fraction *Wg* of OHGs in water and in ethanol plotted against n_C.

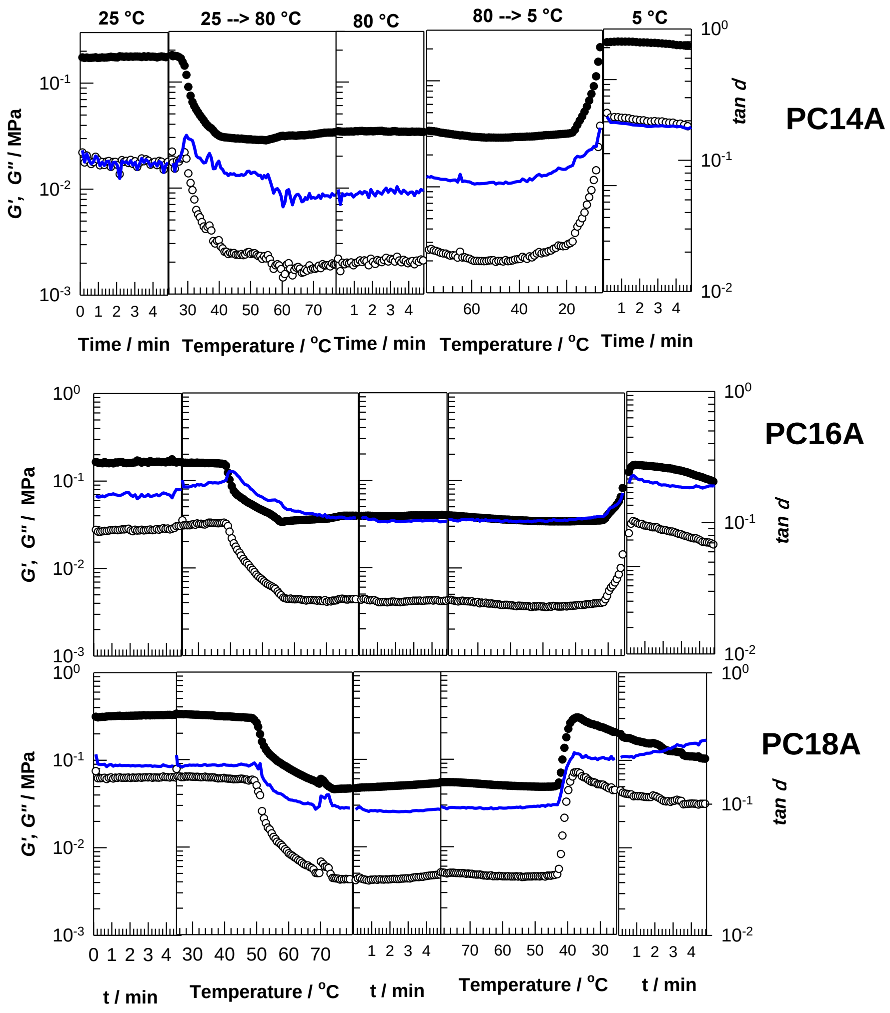

Figure S2. Storage modulus *G'* (filled symbols), loss modulus *G''* (open symbols), and loss factor *tan* δ (lines) of OHGs containing PC14A, PC16A, and PC18A micro-inclusions subjected to a thermal cycle between 25 and 65 °C. γ_o = 0.1%. ω = 6.28 rad·s^{-1}.

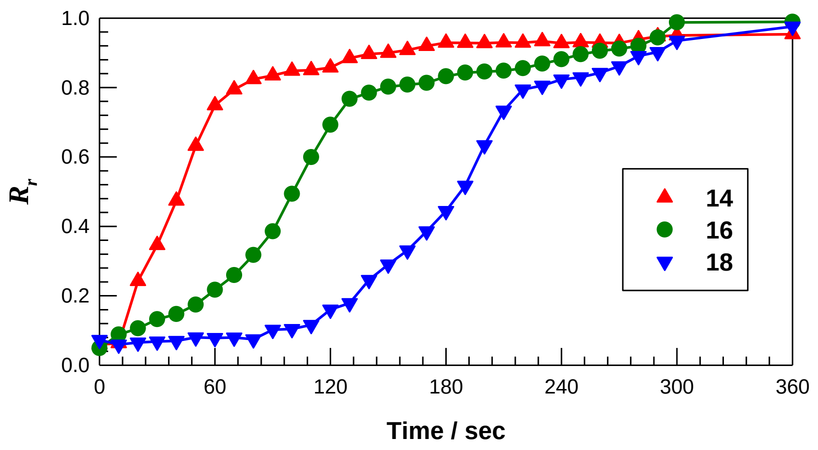

Figure S3. Time-dependent shape-recovery ratio R_r of OHG's with various n_C as indicated. Temperature = 65 °C

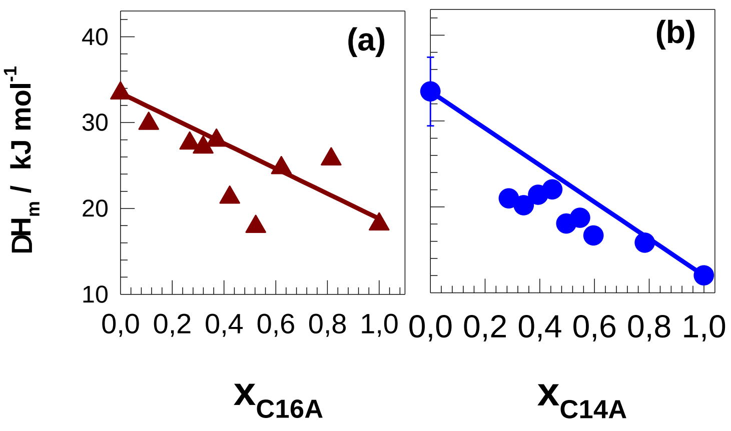

Figure S4. Composition dependence of the melting enthalpies ΔH_m of P(C18A-co-C16A) (a) and P(C18A-co-C14A) copolymers (b). The solid lines are predicted values using the equation $\Delta H_m = \sum \Delta H_{m,i}\, x_i$, where $\Delta H_{m,i}$ and x_i are melting enthalpy and the mole fraction of component i in the copolymer, respectively.

References

(S1) Oral, C. B.; Yetiskin, B.; Cil, C.; Kok, F. N.; Okay, O. Silk Fibroin-Based Shape-Memory Organohydrogels with Semicrystalline Microinclusions. *ACS Appl. Bio Mater*. **2023**, 6, 1594−1603.

(S2) Oral, C. B.; Yetiskin, B.; Okay, O. Stretchable silk fibroin hydrogels. *Int. J. Bio. Macromol.* **2020**, 161, 1371-1380.

(S3) Argun, A.; Can, V.; Altun, U.; Okay, O. Non-ionic Double and Triple Network Hydrogels of High Mechanical Strength. *Macromolecules* **2014**, 47, 6430-6440.
